# Effect of Preoperative Intravitreal Bevacizumab on the Surgical Outcome of Neovascular Glaucoma at Different Stages

**DOI:** 10.1155/2017/7672485

**Published:** 2017-06-20

**Authors:** Junki Kwon, Kyung Rim Sung

**Affiliations:** Department of Ophthalmology, College of Medicine, University of Ulsan and Asan Medical Center, Seoul, Republic of Korea

## Abstract

**Purpose:**

To evaluate the effect of preoperative intravitreal bevacizumab (IVB) injection on the surgical outcome of Ahmed glaucoma valve (AGV) implantation according to the angle status in neovascular glaucoma (NVG) eyes.

**Materials and Methods:**

This retrospective study included 70 NVG patients who underwent AGV implantation and were followed up for at least 12 months. An IVB injection before AGV implantation was administered to 45 eyes (IVB group), while it was not administered to 25 eyes (control group). Subgroup analyses were done at different stages in terms of the extent of peripheral anterior synechiae (PAS).

**Results:**

Mean follow-up period after AGV implantation was 27 ± 15 months. The IVB group showed higher prevalence of the eyes with less than 50% of PAS than that of the control group (78% versus 44%). The overall success rate 1 year postoperatively was 80% and 64% for the IVB and control groups, respectively (*P* = 0.142). When PAS extent was less than 50%, preoperative IVB had a marginally positive effect on surgical outcome (HR = 0.39, *P* = 0.064, per 1-time IVB injection).

**Conclusions:**

Preoperative IVB may enhance the success rate of AGV implantation in NVG eyes, before PAS has extensively formed. Further prospective randomized studies controlling the extent of PAS are warranted.

## 1. Introduction

Neovascular glaucoma (NVG) is still a medical and surgical challenge for ophthalmologists. The main causes of NVG are ischemic retinal conditions, such as proliferative diabetic retinopathy (PDR), central retinal vein occlusion (CRVO), and ocular ischemic syndrome (OIS) [1]. New vessels (NV) that are formed at the iris and anterior chamber angle and contracture of the fibrovascular membrane at the angle result in progressive angle closure and intraocular pressure (IOP) elevation [2]. The principle reason of IOP elevation is secondary angle closure due to peripheral anterior synechiae (PAS) [2].

Several angiogenic factors are involved in the neovascularization of the anterior segment in NVG [1]. Among them, the role of vascular endothelial growth factor (VEGF) type A has been well characterized in the pathogenesis of NVG [3, 4]. Since a significantly increased level of VEGF was detected in the aqueous humor in the eyes of patients with NVG [4], treatment that particularly targets this angiogenic factor has emerged. Bevacizumab (Avastin; Genentech Inc., South San Francisco, CA), which is a monoclonal antibody that was approved for treatment of metastatic colon cancer in 2004 by the US Food and Drug Administration, has also been used to treat NVG. Many reports have been published about the effect of intravitreal bevacizumab (IVB) injection on NVG [5–14]. Adjuvant IVB injection may lead to regression of NV in the iris and angle, thus, reducing the incidence of hyphema and potentially enhancing the surgical outcome of Ahmed glaucoma valve (AGV) implantation in NVG [10, 12]. However, its role in treating NVG is currently limited. Further, IVB showed no significant effect on the long-term surgical outcome in some publications [5, 9, 10, 12].

The effect of IVB on medical treatment of NVG at different stages was previously reported in 2008 [15]. This study reported that while IVB might stabilize iris NV and control IOP in patients with early-stage open-angle NVG, it could not control IOP in advanced angle-closure NVG. One of the limitations of this previous study, as noted by the authors, was the absence of a control group [15].

In the present study, we compared the surgical outcomes of the eyes of patients with NVG, in terms of IOP between those who received a preoperative IVB injection with AGV implantation (IVB group) and those who underwent AGV implantation alone (control group). Additionally, we performed subgroup analysis based on the anterior chamber angle status (i.e., the extent of PAS) to assess the adjuvant effect of preoperative IVB on the outcome of AGV implantation at different stages of NVG.

## 2. Materials and Methods

### 2.1. Subjects

The medical records of patients who underwent AGV implantation for the treatment of NVG from Nov. 2010 to Dec. 2015, by a single surgeon (KRS) at Asan Medical Center, were retrospectively reviewed. NVG was diagnosed by neovascularization of the iris and/or iridocorneal angle, with an IOP of more than 21 mmHg by Goldmann applanation tonometry (GAT) [12]. Among them, patients with a follow-up period of less than 1 postoperative year, younger than 18 years, and who had undergone previous glaucoma surgery, including a cyclodestructive procedure, were excluded. Further, patients with no preoperative information about detailed angle status (assessed using gonioscopy) were excluded. Subgroups were categorized according to anterior chamber angle status, with a cut-off point of 50% PAS (less than 50% versus more than 50%), as assessed by gonioscopy using a Sussman lens. This study was approved by the institutional review board of Asan Medical Center, Seoul, Korea, and conformed the tenets of the Declaration of Helsinki.

### 2.2. Treatment Plan of Neovascular Glaucoma

After diagnosis of NVG, IVB injection was done as an outpatient procedure with topical anesthesia. After draping with 5% povidone iodine solution, 0.05 cc of bevacizumab (25 mg/cc) was injected intravitreally via the pars plana, approximately 3.5 mm from the limbus, with a 30-gauge needle. After injection, visual acuity (VA) was tested to verify the perfusion of the optic nerve. If required, anterior chamber paracentesis was done to prevent excessive IOP elevation. Topical prophylactic antibiotics (moxifloxacin or gatifloxacin) were prescribed 4 times per day, for 3–5 days after IVB injection. Repeated injections of IVB were performed at the discretion of the glaucoma specialist, if required.

Panretinal photocoagulation (PRP) was done before AGV implantation for the majority of patients included in this study, except where there was severe media opacity in the eye, such as corneal edema, dense cataract, hyphema, and/or vitreous hemorrhage. PRP was administered to 38 (84%) of the 45 eyes in the IVB group and to 18 (72%) of 25 eyes in the control group (*P* = 0.212 by chi-square test).

AGV implantation was performed by a single glaucoma specialist (KRS) using a standardized surgical technique. The Ahmed-FP7 (New World Medical Inc., CA) model was used for all eyes. Initially, a subconjunctival lidocaine injection was administered, either supratemporally or supranasally. A corneal traction suture was then performed, before a fornix-based conjunctival pocket was made (either supratemporally or supranasally). Although the supratemporal site was preferred, the sites were chosen according to the surgeon's discretion, depending on factors, such as conjunctival scarring, presence of peripheral anterior synechiae, and depth of the anterior chamber, which affected the entry of the tube into the anterior chamber. To prevent excessive postoperative fibrosis, vessels were not cauterized during conjunctival dissection and scleral flap formation. After exposure of the scleral bed, measuring about 5 × 7 mm, a limbal-based partial-thickness scleral flap was prepared using a Beaver blade. The AGV was primed with balanced saline solution to confirm patency and a polypropylene (5-0 Prolene) thread was incorporated into the tube lumen. The tube was then ligated near the tube-plate junction with an absorbable polyglactin (8-0 Vicryl) suture, and the 5-0 Prolene thread was removed. The plate was placed on the sclera about 8–10 mm behind the limbus and was secured to the sclera with 9-0 nylon. The tube was trimmed to an appropriate length and inserted into the anterior chamber through a 23-gauge needle tract beneath the scleral flap. The tube was then fixed on the sclera with a 9-0 nylon suture, and the scleral flap was closed with two 9-0 nylon sutures. Finally, the conjunctiva was reapproximated to the limbus with an 8-0 Vicryl. No subconjunctival injection of antibiotics or dexamethasone was given. Corticosteroid ointment and a pressure patch were applied at the end of the surgery. Postoperatively, topical antibiotics, steroid, and atropine were administered 2–4 times per day for 4 weeks, 4–8 times per day for 4 weeks, and 2 times per day for 2 weeks, respectively.

### 2.3. Data Collection

The following preoperative baseline data were collected: age, gender, the cause of NVG (e.g., PDR, CRVO, and OIS), IOP, best-corrected VA (BCVA, which was measured using the Log minimal angle of resolution [LogMAR]), presence of NV at angle, the extent of PAS, and follow-up period after AGV implantation. The main outcome measures were IOP and BCVA at final visits. Surgical success was defined as an IOP between 6 and 21 mmHg, without loss of light perception (LP), and with or without the use of antiglaucoma medication [12]. Surgical failure was defined as an IOP of more than 21 mmHg or less than 6 mmHg at two consecutive follow-up visits, the loss of LP, or a need for additional glaucoma interventions [12]. A significant change in the VA was defined as a change of two or more Snellen line VA, or a change in category (e.g., count fingers to hand motions) after surgery [10]. For statistical analysis, we used a LogMAR value of 2.6 to represent vision of counting fingers and used extrapolated LogMAR values of 2.7, 2.8, and 2.9 to represent hand motion, light perception, and no light perception, respectively [16]. One was randomly chose if both eyes were eligible.

### 2.4. Statistical Analysis

Data were presented as mean ± standard deviation for continuous variables or as numbers with percentages for categorical variables. Comparisons between the groups were done using an unpaired *t*-test, Mann–Whitney *U* test, chi-square test, and Fisher's exact test, as appropriate. A Cox proportional hazard analysis was done to assess risk factors for surgical failure. For subgroups that were categorized according to the extent of PAS, comparisons of surgical outcomes between the IVB group and the control group were done separately. A Cox proportional hazard analysis was also performed for each subgroup. For Cox proportional hazard analysis, independent variables with *P* < 0.10 in univariate analysis were selected and entered for multivariate analysis. All statistical analyses were performed using the SPSS version 18.0 (SPSS, Chicago, IL, USA) statistical package.

## 3. Results

A total of 70 patients met the inclusion criteria and included in the final analysis, of which a preoperative IVB injection was administered to 45 eyes (IVB group) and was not administered to 25 eyes (control group). For the IVB group, an average of 1.56 (maximum value of 4) preoperative IVB injections was administered to each eye. All patients were Korean. The mean age was 58 ± 13 years, and mean follow-up period after AGV implantation was 27 ± 15 months. The main causes of the NVG were PDR for 47 cases (67%), CRVO for 8 cases (11%), and OIS for 12 cases (17%).  Table 1 summarizes baseline characteristics for both groups. There was no significant difference in age, gender, cause of NVG, baseline IOP, BCVA, and follow-up period between the two groups. However, there was significant difference in the extent of PAS between the two groups. The proportion of the eyes where the extent of PAS of more than 50% was greater in the control group (22% versus 56%; *P* = 0.004 by chi-square test).

Table 2 summarizes changes in the IOP and BCVA after AGV implantation in both groups. No significant differences were found between the two groups, in terms of the IOP measured at 1 year postoperatively and at final visit, BCVA at the final visit, or changes in the BCVA. Subgroup analysis according to PAS extent also showed no significant differences between the two groups.

**Figure 1:**
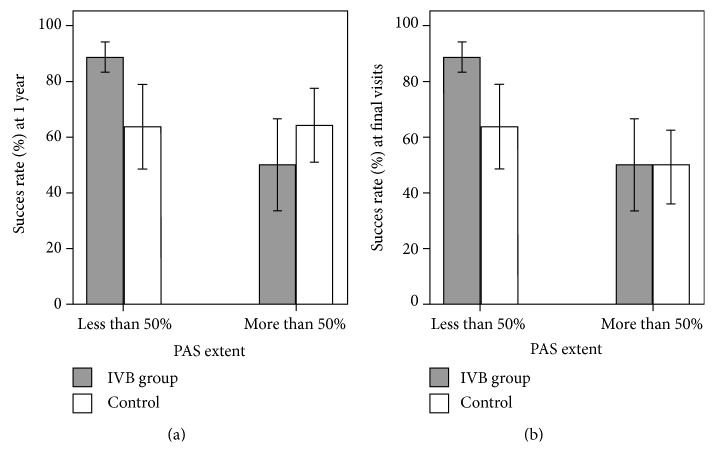
Graphs showing the success rates (with standard error) of glaucoma surgery, (a) at postoperative year 1 and (b) at final visits, between the preoperative intravitreal bevacizumab (IVB) group and the control group, according to the extent of peripheral anterior synechiae (PAS).

The success rates after 1 year and at the final visit are summarized in Table 3 and Figure 1. For all the eyes that were analyzed, the success rate at the final visit was 78% for the IVB group and 56% for the control group, which was marginally significant (*P* = 0.057 by chi-square test). In the subgroup where the extent of PAS was less than 50%, the success rate 1 year postoperatively was 89% in the IVB group and 64% in the control group (*P* = 0.057). In the subgroup where the extent of PAS was more than 50%, the success rates 1 year postoperatively and at final visit were similar between the two groups. The differences between the IVB group and the control group tend to be larger in the subgroup where the extent of PAS was less than 50%, which suggests that IVB plays a greater role during early stages of NVG (Figure 1).

The risk factors for surgical failure were assessed by a Cox proportional hazard analysis for all the eyes (Table 4). The univariate model indicated that PRP, PAS extent, and IVB had possible associations (*P* < 0.10). When these variables were entered into a multivariate model with backward elimination approach, only PRP and the extent of PAS showed marginal significance (hazard ratio [HR] = 0.40 and 2.25, *P* = 0.072 and 0.075, resp.). For the subgroup where the extent of PAS was less than 50%, the IVB injection showed a protective effect of surgical failure, with a HR of 0.39 and borderline significance (*P* = 0.064, per 1-time IVB injection) (Table 5). For the eyes where the extent of PAS was more than 50%, the IVB group (compared to the control group) had no significant association with surgical failure (*P* = 0.775).

## 4. Discussion

Traditional management of NVG includes antiglaucoma medications, PRP, glaucoma filtering surgery, and/or drainage device implantation [10]. Despite these modalities, NVG is still difficult to manage and sometimes results in devastating visual loss. Therefore, early diagnosis of NVG and proper treatment to minimize visual loss and control IOP are essential. The main mechanism of NVG is retinal ischemia [5]. PRP ablates the ischemic retina to decrease tissue oxygen demand, thereby reducing VEGF release and the formation of NV [17]. When delivered before IOP elevation, PRP can reduce the incidence of NVG in ischemic ocular conditions [17]. Therefore, PRP is the essential treatment for NVG. However, it is often difficult to administer laser treatment to the eyes of patients with NVG that have a presence of cloudy media due to corneal edema that results from elevated IOP, hyphema, and/or vitreous hemorrhage. In our study, PRP was given to a total of 80% of the eyes before AGV implantation. More eyes in the IVB group underwent PRP than those in the control group (84% versus 72%), but there was no statistically significant difference (*P* = 0.212).

Recently, anti-VEGF agents have emerged as adjuvant therapy for retinal ischemia, since the level of VEGF is increased in the aqueous or vitreous of the eyes of patients with NVG [4, 18]. Angiogenic factors, including VEGF, promote the creation of fibrovascular membranes, which lead to an increase in IOP. Boyd et al. [19] reported that since the level of VEGF in aqueous had a temporal correlation with the course of NV formation in eyes of patients with ischemic CRVO, anti-VEGF treatment during the early stages of NGV might be therapeutically beneficial. Bevacizumab, an anti-VEGF monoclonal antibody, has been widely used as off-label to treat neovascular age-related macular degeneration, diabetic macular edema, and CRVO [5]. The half-time of a single dose (0.05 cc) of the IVB injection is about eight to nine days in the human eyes [20, 21]. Therefore, the effect of IVB may be transient and repeated injections are sometimes needed [15]. In our study, as many as 4 times, IVB injections were performed in the eyes of the IVB group (mean 1.56 times).

There have been many studies reporting the effect of bevacizumab in the eyes of patients with NVG [5–15, 17]. Wakabayashi et al. [15] retrospectively reviewed 41 patients with different stages of NVG, including iris NV without elevated IOP, open-angle NVG, and angle-closure NVG. In that study, all patients received IVB as an initial treatment, which showed no effect on controlling IOP and stabilizing NV, except for patients with angle-closure NVG [15]. Another study by Sahyoun et al. [12], which compared the surgical outcomes of NVG patients who did and did not receive preoperative IVB, reported that although preoperative IVB was not associated with a better surgical success, IOP control, or VA, it significantly decreased postoperative hyphema and was associated with a requirement for fewer antiglaucoma medications. In 2015, Arcieri et al. [10] reported a prospective, randomized clinical trial on the efficacy of concomitant and postoperative IVB injection with AGV implantation, compared with AGV implantation alone. They concluded that although there was no difference in the survival success rates between the two groups, the IVB group required fewer antiglaucoma medications and showed more frequent regression of iris NV [10]. Another retrospective, comparative study revealed that IVB had no long-term effects on patients with NVG and had a limited, temporizing role in the treatment of NVG [5].

A substantial portion of the eyes of patients with NVG did not respond to medical therapy with antiglaucoma medication and ultimately required surgical treatment. The success rate of conventional filtering surgery is relatively low for patients with NVG [12]. A drainage implant, such as an AGV, is of particular use in these conditions and is less likely to fail in comparison to trabeculectomy [5]. To the best of our knowledge, a comparative study on the efficacy of IVB in patients with NVG at different stages of the disease has not been previously published. Therefore, in the current study, we retrospectively reviewed medical records of patients, who consecutively underwent AGV implantation for the treatment of NVG, and enrolled those with detailed angle description, which was assessed by a glaucoma specialist using gonioscopy. Our study comprised a relatively large number of eyes of patients with NVG (70 eyes in total) and a long follow-up period, with an average of 27 months. Due to the relatively short half-life of intraocular bevacizumab, repeated preoperative injections of IVB were performed as needed, with an average of 1.70 times for the eyes where the extent of PAS were less than 50% and 1.51 times for those where PAS was more than 50% (*P* = 0.481). Although not statistically significant, IVB showed more adjuvant positive effect on surgical success rates at postoperative 1 year and final visits when PAS had not formed extensively, as illustrated in Figure 1.

We also assessed risk factors for surgical failure using a Cox proportional hazard analysis. In univariate analysis, PRP, PAS, and IVB were putative factors for all the eyes, while the PRP and PAS were the related factors in a multivariate model with borderline significances. Nakano et al. [8] retrospectively reviewed 181 eyes of patients with NVG and found that angle closure had the greatest effect on an NVG-IOP prognosis, with a HR of 3.059, which is in line with our results. Olmos et al. [5] who reviewed 163 eyes of patients with NVG with a mean follow-up of 12 months reported that PRP was the most important prognostic factor, with its long-lasting antiangiogenic effect. In our study, IVB showed some protective effect against surgical failure in the group where the extent of PAS was lower. One IVB injection had the effect of reducing the risk of surgical failure by 61% (HR = 0.39, *P* = 0.064). Therefore, IVB showed a positive effect on the surgical outcome of NVG, particularly during the early stages of NVG with an open angle.

Our study has some limitations. First, in the process of enrolling participants, the possibility of selection bias should not be ruled out. From the beginning of the study, we focused on the effect of IVB on different stages of NVG, so detailed information of angle status before AGV implantation was required. Additionally, retrospective chart review did not reveal patients with NVG who were excluded from IVB before AGV implantation for any reason, similar to a previous study [5]. Further, angle status after IVB injection and/or AGV implantation was not assessed due to lack of information on chart. Therefore, the direct assessment of the effect on regression of NV was not done. Another limitation is that the amount of laser ablation might be different among subjects, although most patients received PRP as wide as possible except the area of major vessel arcade. Quantitative assessment of PRP (sessions, total of spots) was therefore challenging, and instead, we only checked if the patient received PRP or not. Next, the extent of PAS was different between the 2 groups, showing 78% of the eyes had PAS less than 50% in the IVB group, but only 44% of the eyes had PAS less than 50% in the control group. In other words, a higher proportion of the eyes with PAS less than 50% underwent a preoperative IVB injection than those with PAS more than 50%. So, one might think that the eyes with severe PAS could be tendentiously not treated with IVB and presented a worse HR for surgical outcome. It is difficult to give a clear answer because there was no predetermined indication about IVB injection for the eyes with NVG, and we could not find the reason why IVB injection was not performed in the control group retrospectively. Although there was no statistical significance, the control group showed slightly higher baseline IOP, showed worse baseline BCVA, had more eyes with OIS which was known to have a bad prognosis, and showed lower rate of PRP than the IVB group. In other words, participants with relatively severe stages of NVG and/or media opacity might be more likely to be distributed in the control group. This could lead to a confounding effect in the success rate and Cox proportional hazard analysis to assess the risk factors of surgical failure.

In summary, there was no difference in terms of the final IOP and BCVA between the IVB group and the control group. However, IVB injection before AGV implantation showed the possibility of enhancing the surgical success rate when PAS is still not yet extensively formed. Further prospective, well-designed, and randomized controlled studies assessing the effect of IVB will be warranted.

## Figures and Tables

**Table 1:** Baseline demographics and ocular characteristics of participants (total of 70 eyes).

	Total (*n* = 70 eyes)	IVB group (*n* = 45 eyes)	Control (*n* = 25 eyes)	*P* value (IVB versus control)
Gender (male/female, number of patients)	56/14	38/7	18/7	0.212
Age (years)	58 ± 13	59 ± 10	57 ± 18	0.556
Causes of NVG, number (%)				
PDR	47 (67)	33 (73)	14 (56)	
CRVO	8 (11)	5 (11)	3 (12)	0.110
OIS	12 (17)	7 (16)	5 (20)	
Baseline IOP (mmHg)	40.5 ± 9.2	40.3 ± 9.7	41.0 ± 8.4	0.759
Baseline BCVA (LogMAR)	2.01 ± 1.11	1.96 ± 1.07	2.12 ± 1.20	0.556
Presence of NVA, number (%)	66 (94)	42 (93)	24 (96)	1.000
PAS extent, number (%)				
Less than 50%	46 (66)	35 (78)	11 (44)	**0.004**
More than 50%	24 (34)	10 (22)	14 (56)	
Preoperative PRP, number (%)	56 (80)	38 (84)	18 (72)	0.212
Postoperative follow-up (months) [range]	27 ± 15 [12–68]	26 ± 16 [12–68]	27 ± 14 [12–67]	0.721

IVB = intravitreal bevacizumab; NVG = neovascular glaucoma; PDR = proliferative diabetic retinopathy; CRVO = central retinal vein occlusion; OIS = ocular ischemic syndrome; IOP = intraocular pressure; BCVA = best-corrected visual acuity; MAR = minimal angle of resolution; NVA = neovascularization of angle; PAS = peripheral anterior synechiae; PRP = panretinal photocoagulation. Continuous variables are represented as mean ± standard deviation.

**Table 2:** Changes of intraocular pressure and best-corrected visual acuity after Ahmed glaucoma valve implantation in the 2 groups.

Total	IVB group (*n* = 45 eyes)	Control (*n* = 25 eyes)	*P* value

Baseline IOP (mmHg)	40.3 ± 9.7	41.0 ± 8.4	0.759
PO 1-year IOP (mmHg)	15.3 ± 3.7	15.6 ± 2.8	0.660
Final IOP (mmHg)	15.5 ± 4.2	16.6 ± 4.4	0.290
Final BCVA (LogMAR)	1.62 ± 1.19	1.92 ± 1.29	0.334
BCVA increased	16 (36)	8 (32)	0.320
BCVA unchanged	20 (44)	8 (32)	
BCVA decreased	9 (20)	9 (336)	

PAS less than 50%	IVB group (*n* = 35 eyes)	Control (*n* = 11 eyes)	*P* value

Baseline IOP (mmHg)	40.7 ± 9.6	38.6 ± 8.1	0.497
PO 1-year IOP (mmHg)	15.5 ± 3.5	15.6 ± 2.2	0.958
Final IOP (mmHg)	15.7 ± 4.1	15.6 ± 2.2	0.880
Final BCVA (LogMAR)	1.51 ± 1.17	0.91 ± 1.14	0.140
BCVA increased	14 (40)	5 (46)	1.000
BCVA unchanged	14 (40)	4 (36)	
BCVA decreased	7 (20)	2 (18)	

PAS more than 50%	IVB group (*n* = 10 eyes)	Control (*n* = 14 eyes)	*P* value

Baseline IOP (mmHg)	38.7 ± 10.3	42.9 ± 8.4	0.281
PO 1-year IOP (mmHg)	14.5 ± 4.4	15.7 ± 3.3	0.447
Final IOP (mmHg)	14.5 ± 4.4	17.4 ± 5.6	0.182
Final BCVA (LogMAR)	2.00 ± 1.25	2.71 ± 0.73	0.128
BCVA increased	2 (20)	3 (21)	0.273
BCVA unchanged	6 (60)	4 (29)	
BCVA decreased	2 (20)	7 (50)	

IVB = intravitreal bevacizumab; IOP = intraocular pressure; PO = postoperative; BCVA = best-corrected visual acuity; MAR = minimal angle of resolution; PAS = peripheral anterior synechiae. Continuous variables are represented as mean ± standard deviation and categorical variables as number (%).

**Table 3:** Success rates (%) after Ahmed glaucoma valve implantation in the 2 groups for total participants and subgroups according to extent of peripheral anterior synechiae.

Total (*n* = 70 eyes)	IVB group	Control	*P* value

PO 1 year	80 (36/45)	64 (16/25)	0.142
Final visits	78 (35/45)	56 (14/25)	0.057

PAS less than 50% (*n* = 46 eyes)	IVB group	Control	*P* value

PO 1 year	89 (31/35)	64 (7/11)	0.057
Final visits	86 (30/35)	64 (7/11)	0.107

PAS more than 50% (*n* = 24 eyes)	IVB group	Control	*P* value

PO 1 year	50 (5/10)	64 (9/14)	0.678
Final visits	50 (5/10)	50 (7/14)	1.000

IVB = intravitreal bevacizumab; PO = postoperative; PAS = peripheral anterior synechiae.

**Table 4:** Result of cox proportional hazard analysis for assessing risk factors of surgical failure (total of 70 eyes).

Variables	Univariate		Multivariate	
	HR (95% CI)	*P* value	HR (95% CI)	*P* value
Preoperative PRP (versus not done)	**0.38 (0.14–1.02)**	**0.056**	0.40 (0.15–1.09)	0.072
Presence of NVA (versus absence)	0.91 (0.20–4.20)	0.907		
PAS more than 50% (versus less than 50%)	**2.33 (0.96–5.62)**	**0.061**	2.25 (0.92–5.47)	0.075
IVB group (versus control)	**0.47 (0.20–1.11)**	**0.085**		
Number of IVB injection (per 1-time injection)	**0.60 (0.35–1.03)**	**0.062**		
Baseline IOP (per 1 mmHg)	1.03 (0.99–1.08)	0.144		
Baseline BCVA (per 1 LogMAR)	1.23 (0.78–1.93)	0.368		

HR = hazard ratio; CI = confidence interval; PRP = panretinal photocoagulation; NVA = neovascularization of angle; PAS = peripheral anterior synechiae; IVB = intravitreal bevacizumab; IOP = intraocular pressure; BCVA = best-corrected visual acuity; MAR = minimal angle of resolution. Variables with *P* < 0.10 in univariate analysis (PRP, PAS extent, IVB, and number of IVB injection) were entered into multivariate analysis. Multivariate model using a backward elimination approach based on likelihood ratio; variables were entered in the model if *P* < 0.05 and removed if *P* > 0.10 in the saturated multivariate model.

**Table 5:** Result of cox proportional hazard analysis for assessing risk factors of surgical failure at different stages of peripheral anterior synechiae.

Variables	PAS less than 50% (*n* = 46 eyes)				PAS more than 50% (*n* = 24 eyes)	
	Univariate		Multivariate		Univariate	
	HR (95% CI)	*P* value	HR (95% CI)	*P* value	HR (95% CI)	*P* value
Preoperative PRP (versus not done)	0.34 (0.08–1.37)	0.130			0.51 (0.12–2.13)	0.354
Presence of NVA (versus absence)	0.39 (0.08–1.90)	0.242			NA^∗^	
IVB group (versus control)	**0.29 (0.08–1.10)**	**0.069**			1.19 (0.37–3.81)	0.775
Number of IVB injection (per 1-time injection)	**0.39 (0.15–1.06)**	**0.064**	0.39 (0.15–1.06)	0.064	0.91 (0.51–1.61)	0.743
Baseline IOP (mmHg)	1.05 (0.98–1.13)	0.186			1.02 (0.95–1.09)	0.624
Baseline BCVA (LogMAR)	1.05 (0.89–1.88)	0.881			1.25 (0.56–2.78)	0.582

^∗^All 24 eyes had NVA. HR = hazard ratio; CI = confidence interval; NVA = neovascularization of angle; PAS = peripheral anterior synechiae; IVB = intravitreal bevacizumab; IOP = intraocular pressure; BCVA = best-corrected visual acuity; MAR = minimal angle of resolution; NA = nonapplicable. Variables with *P* < 0.10 in univariate analysis were entered into multivariate analysis. Multivariate model using a backward elimination approach based on likelihood ratio; variables were entered in the model if *P* < 0.05 and removed if *P* > 0.10 in the saturated multivariate model.
